# Three-Year Follow-Up after Intrauterine mTOR Inhibitor Administration for Fetus with TSC-Associated Rhabdomyoma

**DOI:** 10.3390/ijms241612886

**Published:** 2023-08-17

**Authors:** Anita Maász, Tímea Bodó, Ágnes Till, Gábor Molnár, György Masszi, Gusztáv Labossa, Zsuzsanna Herbert, Judit Bene, Kinga Hadzsiev

**Affiliations:** 1 Department of Medical Genetics, Medical School and Clinical Centre, University of Pécs, H-7624 Pécs, Hungary; 2 Bethesda Children’s Hospital, H-1146 Budapest, Hungary; 3 Department of Obstetrics and Gynaecology, Medical School and Clinical Centre, University of Pécs, H-7624 Pécs, Hungary; 4 Department of Paediatrics, Medical School and Clinical Centre, University of Pécs, H-7624 Pécs, Hungary; 5 Department of Medical Imaging, Medical School and Clinical Centre, University of Pécs, H-7624 Pécs, Hungary

**Keywords:** rhabdomyoma, tuberous sclerosis complex (TSC), everolimus, *TSC*2, follow-up

## Abstract

Tuberous sclerosis complex (TSC) is a multisystem disorder characterized by seizures, neuropsychiatric disorders, and tumors of the heart, brain, skin, lungs, and kidneys. We present a three-year follow-up of a patient with TSC-associated rhabdomyoma detected in utero. Genetic examination of the fetus and the parents revealed a de novo variant in the *TSC2* gene (c.3037delG, p.Asp1013IlefsTer3). Oral everolimus was initiated in the pregnant mother to regress the fetal tumor, which was successful. To the best of our knowledge, there is very little information regarding the use of everolimus therapy during pregnancy. West-syndrome was diagnosed when the proband was four months old. The symptoms were well-manageable, however temporarily. Therapy-resistant focal seizures were frequent. The patient had good vitals and was under regular cardiological control, showed a balanced circulation, and did not require any medication. Subependymal giant cell astrocytoma (SEGA) identified by regular neuroimaging examinations remained unchanged, which may be a consequence of early intrauterine treatment. Early detection of the pathogenic *TSC2* variant, followed by in utero administration of everolimus and early vigabatrin therapy, allowed the detection of a milder developmental delay of the proband. Our study emphasizes how early genetic testing and management of epilepsy are pivotal for proper neurodevelopmental impacts and therapeutic strategies.

## 1. Introduction

Tuberous sclerosis complex (TSC) is a rare autosomal dominant disorder. The birth incidence is estimated to be one in 6000–10,000 births [1,2]. In Hungary, the disorder affects approximately 15 infants annually; however, most cases remain unrecognized. TSC is characterized by the development of malignant tumors and hamartomas in multiple organ systems, typically in the brain, eyes, lungs, kidneys, skin and heart [3,4]. The most common manifestations of hamartomas are neurological, renal, and dermatological abnormalities [3]. Other hamartomas, such as cardiac rhabdomyomas, are also associated with TSC in 50–70% of cases [4,5,6]. These tumors can show a large variability in number, size, and location. Most cases are asymptomatic; however, they rarely cause a wide spectrum of disturbances in heart function, such as arrhythmia, cardiomyopathy, and ventricular dysfunction [7]. Rhabdomyomas can develop during pregnancy and even during the second trimester. Fetal forms of rhabdomyomas are rare, with an incidence rate of 0.08–0.27%. The very low incidence rate may be explained by their diagnostic difficulty, as a result of which many cases go unrecognized [8]. Cardiac rhabdomyoma is a known marker for the detection of TSC in utero and may be the only manifestation of the disease for months. Most rhabdomyomas are hormone-dependent tumors that tend to stagnate and regress spontaneously during the first year of life [9].

It is well known that there is no treatment available for TSC; however, there are options for effective symptomatic treatment that can improve the symptoms and delay their worsening (e.g., vigabatrin and cannabidiol for TSC-associated epilepsy). Everolimus (Afinitor^®^, RAD-001 (40-O-(2-hydroxyethyl)-rapamycin)) is a rapamycin analog immunosuppressant and a mammalian target of rapamycin (mTOR) inhibitor that has been administered orally in cases with TSC-related subependymal giant cell astrocytoma (SEGA) and TSC-associated renal angiomyolipoma [10,11]. Several studies have provided evidence of the effectiveness of everolimus in disease progression such as epileptogenesis via the regulation of neuronal death and neurogenesis and the modulation of synaptic plasticity [7,10,11]. However, there is a need for additional clinical studies on everolimus therapy [12,13,14].

Several variants of *TSC1* or *TSC2* have been identified as the cause of TSC. The *TSC2* gene is located on chromosome 16p13.3 and encodes the tuberin protein [15]. This protein assembles a heterodimer complex with hamartin, which is encoded by *TSC1* on chromosome 9q34.13 [16]. Tuberin and hamartin are GTPase-activating proteins (GAP) [17]. The functional TSC1–TSC2 heterodimer complex plays a crucial role in inhibiting the mTOR signaling pathway [18]. Therefore, this complex can influence cell growth, cell proliferation, protein synthesis and metabolism. Recent studies have shown that a third protein, TBC1D7, is also involved in the formation of the TSC-heterodimer through the stabilization of dimerization of the TSC1 C-terminal coiled coil region [19,20]. Variants in either member of the complex can cause impairments in the regulation of the mTOR signaling pathway, which can result in uncontrolled cell proliferation, cell growth, and the development of multisystemic disorders and tumors, especially in the brain, skin, heart and lung [21]. In the literature, more than 1000 *TSC2* and almost 600 *TSC1* pathogenic/likely pathogenic variants have been identified in association with the disease; however, several other epigenetic and environmental factors can influence the severity and onset of symptoms [22].

In this study, we present the three-year follow-up of a Hungarian male patient diagnosed in utero as being genetically confirmed with TSC-associated rhabdomyoma. The patient was treated with everolimus during the pregnancy of a healthy mother.

## 2. Case Presentation

**Figure 1:**
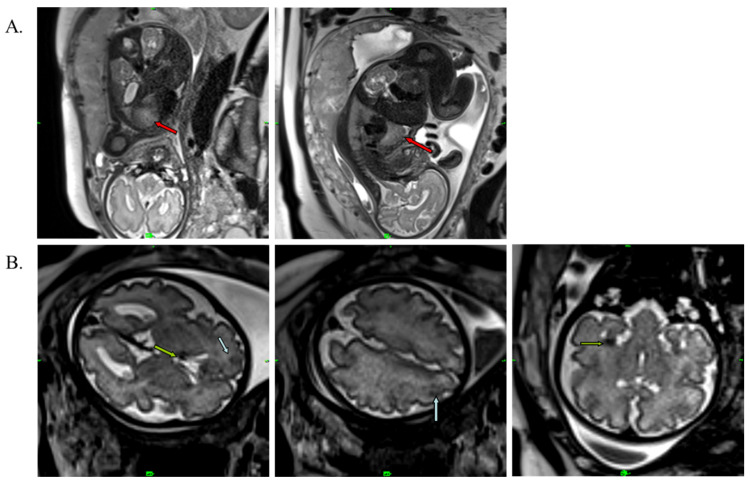
T2-weighted MRI image of the fetus at the 30th week of gestation. (**A**) Red arrows show cardiac rhabdomyoma (31 × 32 × 33 mm). (**B**) Blue arrows show cortical tubers, and green arrows represent subependymal nodules in the brain.

A 34-year-old pregnant woman was referred for examination at the Department of Obstetrics and Gynecology in her 30th week of gestation. The pregnancy was uncomplicated, and fetal parameters, including the abdominal circumference, femur length, and biparietal diameter, were consistent with the gestation age. An abnormal cardiac ultrasound scan of the fetus initiated a fetal echocardiography with a GE Voluson E8 high-end ultrasound system (GE Healthcare, Chicago, IL, USA). Left ventricular expansion was interpreted as a rhabdomyoma (31 × 32 × 33 mm), and some smaller tumors (multiplex tumors) were detected (Figure 1A). The fetal cardiac rhythm was a sinus without arrhythmia. In addition, echo-dense formations (cortical tubers and subependymal nodules) were also described in the brain tissue using fetal magnetic resonance imaging (FMRI), including T2-weighted imaging obtained using HASTE IRM (Siemens Healthcare GmbH, Erlangen, Germany) (Figure 1B).

We performed a transabdominal ultrasound-guided chordocentesis to retrieve a sample of fetal blood for genetic analyses initiated due to suspicion of TSC. The chordocentesis was performed with a 20-gauge needle from the placental origin of the umbilical cord. Pre-test genetic counselling was held, and the examination was carried out in the Department of Medical Genetics, University of Pécs. The parents were non-consanguineous with a negative anamnesis for TSC. Family history was additionally negative for epilepsy, neurological disorders and skin manifestations associated with TSC. The brother of the proband was healthy without any medical conditions mentioned previously. The proband’s mother was at risk for thrombophilia, and she was also treated for hyperthyroidism during the entire pregnancy. Whole peripheral blood samples were taken from both parents. DNA was extracted from the blood of both parents and from umbilical cord blood using E.Z.N.A. Blood DNA Kit according to the protocol suggested by the manufacturer (Omega Biotek, Norcross, GA, USA). Qubit^TM^ 2.0 Fluorometric Quantitation system (Thermo Fisher Scientific Inc., Waltham, MA USA) was used to calculate the quantity and quality of genomic DNA samples.

Firstly, the multiplex ligation-dependent probe amplification (MLPA) method was used to analyze possible large deletions in the *TSC2* gene (NM_000548.5; ENST00000219476.9) according to the protocol of Salsa MLPA Probemix P046 TSC2 (MRC Holland, Amsterdam, The Netherlands). To confirm the positive MLPA results, breakpoint analyses of the deletion were performed by Sanger sequencing on an Applied Biosystems^TM^ 3500 Genetic Analyzer (Thermo Fisher Scientific Inc., Waltham, MA, USA) using BigDye Terminator Chemistry (Thermo Fisher Scientific Inc., Waltham, MA, USA). For direct sequencing of the region of interest, we amplified DNA with specific primers (F: 5′-TTGGCCCTTGGTGATAGGT-3′; R: 5′-CACCCGCTAGGAGGAACTC-3′) (Integrated DNA Technologies Inc., Coralville, IA, USA) using a SensoQuest Labcycler (SensoQuest GmBH, Göttingen, Germany). The NCBI-PRIMER-BLAST tool (NCBI, Bethesda, MD, USA) was used for primer design.

**Figure 2:**
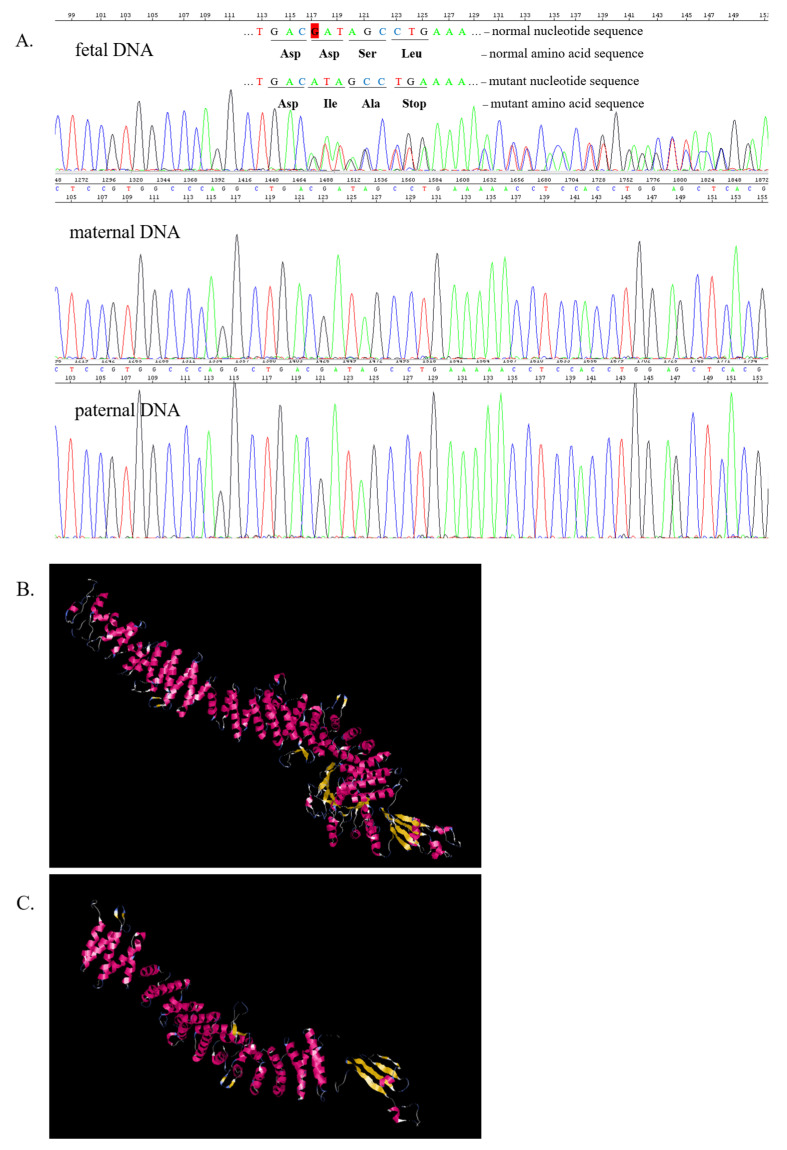
Results of Sanger sequencing of the family and 3D structure of the TSC2 protein. Part (**A**) represents the electropherogram of the proband and shows the *TSC2* c.3037delG, p.Asp1013IlefsTer3 variant. The parents were negative for this variant. Part (**B**) represents the structure of normal TSC2 protein. Part (**C**) shows the TSC2 protein translated from the DNA with c.3037delG.

Sanger sequencing of the sample derived from the umbilical cord revealed a heterozygous frameshift variant in *TSC2* gene in the fetal sample. The novel c.3037delG variant (rs137854301) resulted in the deletion of one G base from the nucleotide sequence and generated an asparaginic acid to isoleucine amino acid change at the 1013 amino acid position and a frameshift leading to a premature stop codon at position 3 in a new reading frame (p.Asp1013IlefsTer3) (Figure 2A). 

The 3D modelling of TSC2 protein, which was carried out using the I-Tasser online server, bioinformatic tool and database [23,24], revealed that the c.3037delG variant in the *TSC2* gene dramatically altered the normal structure of the protein (Figure 2B,C). The sequence variant was interpreted and classified according to the ABC classification system [25] and to the standards and guidelines published by the American College of Medical Genetics and Genomics and Association for Molecular Pathology (ACMG-AMP) [26]. This variant is predicted to cause loss of normal protein function, either through protein truncation (1013–1807) or nonsense-mediated mRNA decay, resulting in an absent or non-functional protein product (PVS1). Other frameshift variants in the nearby region have been reported in individuals with TSC, and loss-of-function of *TSC2* is a well-established mechanism leading to the disorder. This variant has not been observed in the large reference population cohorts of the Genome Aggregation Database (gnomAD, n > 120,000 exomes and n > 15,000 genomes) (PM2) (1000 Genomes Consortium et al., 2015; Exome Variant Server) [27]. To our knowledge, the variant has not been published in the relevant medical literature or reported in the disease-related variation databases, such as HGMD or ClinVar. To assess the impact of the missense change, we applied a combination of some of the most widely used “in silico” computational prediction tools, such as PolyPhen, Provean and MutationTaster [28,29]. These algorithms support obvious evidence that the c.3037delG variant is a protein-damaging, disease-causing variant (PP3). The *TSC2* variant in the parental DNA was normal. Therefore, the variant was assumed to be “de novo” based on the negative family history and negative parental genetic findings (PS2; “with no family history and with confirmation of both maternity and paternity”). To the best of our knowledge, there are no “in vitro” functional studies that investigate the damaging effect of this variant on the gene product (PS3). The classification of this *TSC2* variant provides strong evidence of pathogenicity.

Based on the functional grading of the stepwise ABC system, the variant proved to be a loss-of-function variant that likely has a functional importance (Step A-4). Clinical grading of the *TSC2* c.3037delG variant revealed that the genetic variant has a high penetrance and is linked to the patient’s phenotype as a dominant disorder (Step B-5). Both functional and clinical grades were combined, and a joint class was generated (“Class-B” in Step C), which was linked to the standard comment of the system: “disease-associated pathogenic variant”.

**Figure 3:**
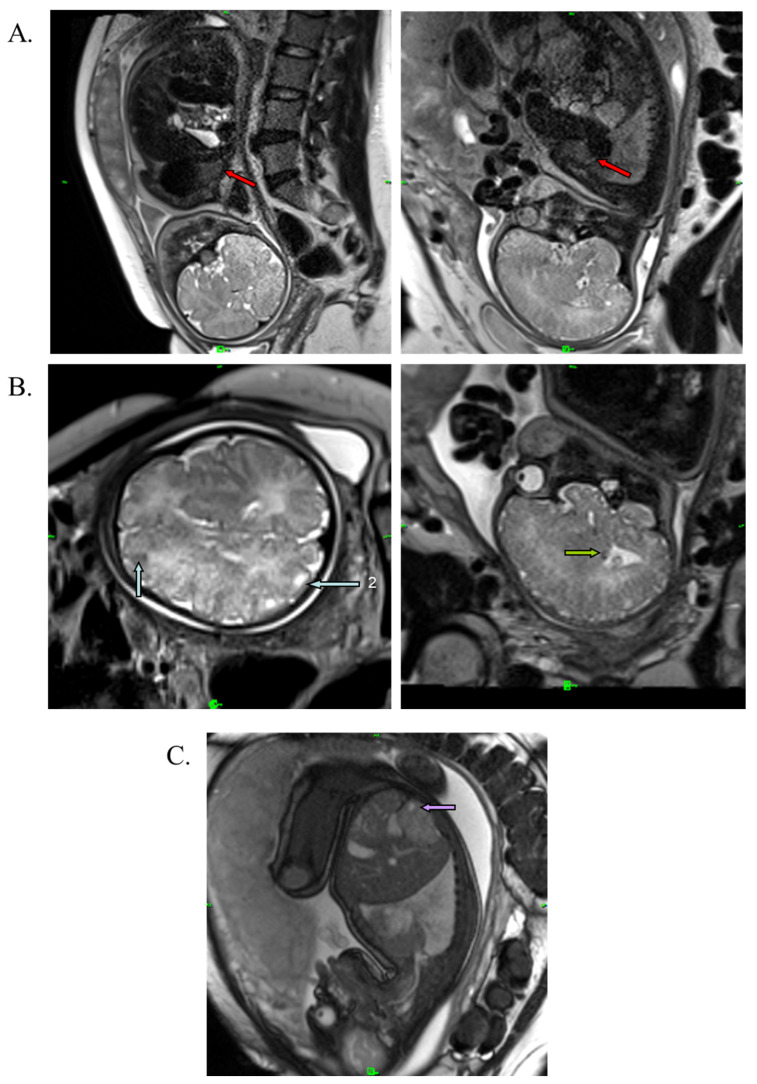
T2-weighted MRI image of the fetus at the 38th week of gestation. (**A**) Red arrows show cardiac rhabdomyoma in reduced size (20 × 20 × 33 mm). (**B**) Blue arrows show cortical tuber in the gyral core (2), and green arrows represent subependymal nodule in the brain. (**C**) Purple arrow shows the renal cyst.

A medical team consisting of a cardiologist, clinical geneticist, obstetrician, and neonatologist decided to start oral immunosuppressant therapy for the mother (everolimus; Votubia). The goal of the treatment was to reduce the size of the rhabdomyoma, which lasted for five weeks. Oral everolimus was given daily. An initial dose of 10 mg/day everolimus was administered. The dose was adjusted to 5 mg/day from day 10 to set the 5–15 ng/mL target trough level (see Table S1). Blood values, including hemoglobin, leukocytes, leukocyte differentiation, thrombocytes, and CRP, were monitored during the treatment. All laboratory parameters were within the normal range. Everolimus reduced the size of the rhabdomyoma by almost half (20 × 20 × 33 mm) (Figure 3A). Everolimus was discontinued on day 36, two weeks before the scheduled cesarean section, to avoid complications and pre- and postnatal infection. The fetus and mother did not experience adverse events during the entire length of the treatment. Fetal MRI performed during the 38th week of gestation also revealed renal cysts in the fetus (Figure 3C).

**Figure 4:**
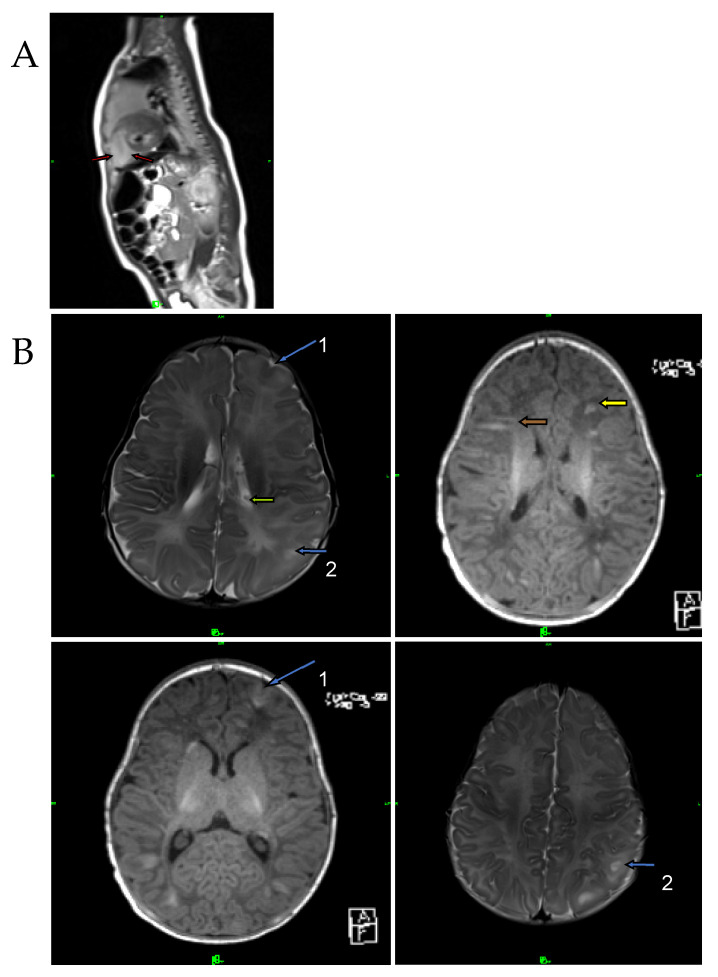
T1-weighted axial MRI image of the brain and T2-weighted sagittal MRI image of the heart at 2 months of age. (**A**) Red arrows represent cardiac rhabdomyoma. (**B**) Blue arrows show cortical tubers in the sulcal island (1) and in the gyral core (2); green arrow represents a subependymal nodule. Brown and yellow arrows show white-matter radial migration lines: straight band (brown), nonspecific conglomerate foci (yellow).

The male infant was born at term by caesarean section with a birth weight of 3700 g (+0.67 SD), birth length of 52 cm (+1.11 SD) and a head circumference of 35 cm (+0.42 SD). This was the mother’s fourth pregnancy (G3, P1). The Apgar score was 9/10. Standard deviations (SD) for birth measurements were calculated using WHO standards [30]. After birth, the boy was monitored in a perinatal intensive care unit for one week. His laboratory parameters were normal. Cardiological examination revealed rhabdomyoma and mitral insufficiency, and the abdominal ultrasound examination suggested angiomyolipoma in the kidneys. The MRI performed at 2 months of age confirmed the findings of the perinatal examinations (Figure 4A). The MRI additionally revealed white-matter radial migration lines, presenting a straight band and nonspecific conglomerate foci (Figure 4B).

The proband has been under close observation by a neurodevelopmental specialist since birth. The proband has shown good growth and development. An EEG was performed at 4 months and detected peaks that corresponded to the left temporal tuber. West’s syndrome was diagnosed at this age based on the following clinical findings: seizures, clinically detected convulsions, infantile spasms observed by the parents, and hypsarrhythmia on EEG. Attention deficit was not observed in the proband. He became seizure-free following vigabatrin and nitrazepam therapy. The proband was under regular cardiological control, had balanced circulation and did not require any medication. At the one-year MRI follow-up, further regression of the tumor was observed. Mild hypotonia was observed during physical examination. Several hypopigmented patches were observed on the abdominal region. We used the Bayley Scales of Infant and Toddler Development (BSID) to measure his physical, cognitive, socioemotional, linguistic and behavioral skills. The results of the BSID at one year of age showed age-appropriate values (TQ > 100). His speech development was on time. The proband had regular cardiological follow-ups and abdominal ultrasound examinations until he was three years old, with normal results. Cranial MRI examination was performed three times during this period, during which the size of the subependymal nodules and SEGA did not change. When the proband was around the age of three, he underwent examination at the neurology department, due to the growing frequency of seizures. The EEG revealed a slow base activity in the left temporal lobe and revealed left temporal epileptic activity. Carbamazepine supplementation was started for the proband. The BSID at three years of age showed a 6–10 months delay in all the parameters (TQ 89–91).

## 3. Discussion

Our case study revealed a de novo pathogenic variant of the *TSC2* gene that has not been previously reported in the medical literature. The substitution of asparaginic acid with isoleucine at position 1013 due to the c.3037delG variant in the *TSC2* gene and the frameshift caused significant changes in the structure, size, and conformation of the TSC2 protein. The ACMG guidelines and ABC classification system provide strong evidence of pathogenicity: this frameshift variant is predicted to cause loss of normal protein function and alteration in its regulatory features through protein truncation [25,26,31]. Based on these predictions, this variant does not affect splice sites; however, the loss of more than 750 amino acids may abolish some crucial features of the TSC2 protein. These features include 5′ adenosine monophosphate-activated protein kinase (AMPK)-mediated phosphorylation and GAP activity. The loss of normal protein function can further inhibit insulin-stimulated phosphorylation and activation of S6K1 protein in the mTORC1-S6K1 pathway. Moreover, this causes a change in its interaction with other interacting proteins, such as TSC1, leading to changes in the TSC1–TSC2 complex [32]. These alterations may result in severe pathological consequences, such as cardiac or renal failure, epilepsy, and other neurodevelopmental and neurobehavioral symptoms, which may lead to a more severe phenotype, onset, and disease state. The disease manifested as a rhabdomyoma in our proband, which is among the major clinical diagnostic criteria of TSC, and it developed early in utero.

In our case report, we observed a regression in the size of the fetal rhabdomyoma due to everolimus administered to the mother during pregnancy. Several previous case reports have described sirolimus as a successful treatment for fetal rhabdomyoma; however, there is very little information about the impact of other rapalogs, such as everolimus. Since 2018, everolimus has been approved by the Food and Drug Administration (FDA) as an adjunctive treatment for adult and pediatric patients aged ≥2 years with TSC-associated partial-onset seizures [33]. It is well known that everolimus is an effective drug for reducing the size of rhabdomyomas and seizure frequency or even for preventing disease progression. Everolimus binds with a high affinity to its cyclophilin receptor, FKBP-12, which in turn binds to the serine/threonine kinase mTOR. This binding forms an mTORC1 complex that includes regulatory-associated protein of mTOR (raptor), mammalian lethal with SEC13 protein 8 (MLST8), proline-rich AKT1 substrate 1 (PRAS40) and DEP domain-containing mTOR-interacting (DEPTOR) proteins. This complex inhibits downstream signaling of mTORC1, which inhibits tumor growth [34]. Studies have observed that patients with TSC are more likely to respond positively to everolimus therapy, independent of the mutation type and site [35]. In the literature, only affected pregnant mothers have been treated directly with everolimus so far [36,37]. In addition, infants with enormous rhabdomyoma detected during prenatal examination were exclusively treated with everolimus after birth [38,39,40,41]. In the literature, it is assumed that everolimus administered to a healthy mother during pregnancy may have a favorable effect on fetal cardiac tumors [42].

Cardiac tumors cause hemodynamic instability, arrhythmias or other complications in most cases [43]. They often produce more severe symptoms in patients; however, the cardiac functions were optimal in our proband, his circulation was always balanced during the follow-up, and he did not require any medication. Manifestations of the disease observed during the three-year follow-up confirmed our assumption of prenatal everolimus therapy decelerating disease progression. Based on our experience during the follow-up, we can state that everolimus administration during pregnancy is safe for both the mother and fetus. The administration of everolimus is highly recommended in similar situations. Further studies are required to confirm these findings.

West syndrome was detected at the age of 4 months in our TSC proband. This syndrome is associated with neurological deficits, intellectual disabilities, autism, and movement disorders that can develop [44]. Adrenocorticotropic hormone (ACTH) and vigabatrin are used for treatment; however, in therapy-resistant forms, other antiepileptic drugs can be used. Vigabatrin can inhibit the gamma-aminobutyric acid (GABA)-degrading enzyme, GABA transaminase, which results in an increased GABA concentration in the brain. Elevated GABA levels can decrease seizures through excitatory processes [45]. In our case, we had an opportunity to administer vigabatrin at an early age due to the early diagnosis of TSC. We observed milder symptoms or the absence of symptoms associated with West syndrome, such as a milder intellectual disability or the absence of autistic features or of developmental delay. These manifestations developed despite treatment in our proband, but symptoms were sufficiently controlled by vigabatrin. A strong correlation can be found between the starting time of therapeutic drugs and the appearance and strength of manifestations in the central nervous system. These symptoms account for the major causes of morbidity and mortality among TSC patients and should not be neglected [3].

## 4. Conclusions

The present case with a novel pathogenic frameshift *TSC2* variant broadens the genetic spectrum of TSC. The timing of genetic tests and interventions can have a crucial impact on the severity of developmental and intellectual disorders and on further therapeutic strategies; therefore, they should be carried out as early as possible to identify persons at high risk. Our case report broadens the current knowledge of the therapeutic possibilities of everolimus and can help medical teams worldwide in clinical decision-making. Our report provides more data on the efficacy and safety of everolimus therapy in fetuses, especially on possible long-term effects. Similar case studies are needed to confirm our assumption on the relationship between disease manifestations and treatment. Our study emphasizes the importance of close monitoring, lifelong multidisciplinary follow-up, and the management of patients with TSC.

## Data Availability

The data presented in this study are available on request from the corresponding author.

## Supplementary Materials

The following supporting information can be downloaded at: https://www.mdpi.com/article/10.3390/ijms241612886/s1.
