# Supplementary material for: Three-Year Follow-Up after Intrauterine mTOR Inhibitor Administration for Fetus with TSC-Associated Rhabdomyoma
Source: Int J Mol Sci. 2023 Aug 17;24(16):12886. doi: 10.3390/ijms241612886 (PMC10454323; doi:10.3390/ijms241612886)
Supplement: Supplementary file 1 [file ijms-24-12886-s001.zip › ijms-2508028-supplementary.pdf]

Table S1. Everolimus dosage.

Days	Doses of everolimus (mg/day)	Serum concentration of everolimus (ng/ml)
1.		3.8*
2.		5.6
3.		6.1
4.		7.0
5.	10	6.9*
6.		7.4
7.		7.6
8.		7.4
9.		7.6*
10.		9.3
11.		8.1
12.		7.5*
16.	5	6.9*
19.		8.3*
23.		8.5*
26.		8.3*
30.		6.5
33.		8.5
37.		3.1
40.		1.5
45.	-	0.9
46.		0.7*[#]
48.		1*
one year after delivery	-	N.A.*

* on these days other laboratory parameters (hemoglobin, leukocytes, leukocyte differentiation, thrombocytes and CRP) were also measured; # date of birth
